# Effect of garlic powder supplementation on blood pressure and hs‐C‐reactive protein among nonalcoholic fatty liver disease patients: A randomized, double‐blind, placebo‐controlled trial

**DOI:** 10.1002/fsn3.2307

**Published:** 2021-05-06

**Authors:** Davood Soleimani, Seyedeh Parisa Moosavian, Hamid Zolfaghari, Zamzam Paknahad

**Affiliations:** ^1^ Nutritional Sciences Department School of Nutrition Sciences and Food Technology Kermanshah University of Medical Sciences Kermanshah Iran; ^2^ Department of Clinical Nutrition School of Nutrition and Food Sciences Isfahan University of Medical Sciences Isfahan Iran; ^3^ Department of Community Nutrition School of Nutritional Sciences and Dietetics Tehran University of Medical Sciences Tehran Iran

**Keywords:** blood pressure, garlic, hs‐CRP, nonalcoholic fatty liver disease

## Abstract

Based on the anti‐inflammatory and antihypertensive properties of garlic, the current study was designed to evaluate the garlic powder effects on blood pressure and high‐sensitivity C‐reactive protein (hs‐CRP) among Nonalcoholic Fatty Liver Disease patients (NAFLD). This randomized, double‐blind, placebo‐controlled trial study was conducted on 110 patients with NAFLD. The patients were randomly divided into 2 groups, receiving two tablets of either 400 mg garlic or placebo daily for 15 weeks. At baseline and the end of the study, blood pressure and hs‐CRP were determined. Of 110 patients enrolled in the trial, 98 subjects were included in the final analysis. After the intervention, systolic blood pressures (SBP) (mean: −7.89; 95%CI:‒11.39 to −4.39 mm Hg), diastolic blood pressure (DBP) (mean: −5.38; 95%CI: −7.77 to −3 mm Hg), and Mean Arterial Pressure (MAP) (mean: −6:95%CI: −8.4 to −3.6 mm Hg) decreased significantly in the garlic group as compared to the placebo group. Also, the percentage of reduced hs‐CRP was significantly higher in the intervention group compared with the control group (mean: −16.1; 95%CI: −32.7 to −0.53; *p* = .035). Moreover, a positive correlation was observed between the percentage change in hs‐CRP and percentage changes in SBP (*r* = 0.221; *p* = .029), DBP (*r* = 0.166; *p* = .012), and MAP (*r* = 0.210; *p* = .038). Garlic supplementation can be a safe and potentially adjunct treatment to reduce blood pressure and the risk of cardiovascular disorders in patients with NAFLD.

## Graphical abstract

This randomized, double‐blind, placebo‐controlled trial study was conducted on 110 patients with NAFLD. The patients were randomly divided in to 2 groups, receiving two tablets of either 400 mg garlic or placebo daily for 15 weeks. After intervention, systolic blood pressures (SBP), diastolic blood pressure (DBP), and Mean Arterial Pressure (MAP) decreased significantly in the garlic group as compared to the placebo group. Also, the percentage of reduced hs‐CRP was significantly higher in the intervention group compared with the control group. Moreover, a posetive correlation was observed between percentage change in hs‐CRP and percentage changes in SBP, DBP, and MAP.

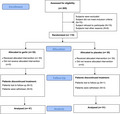

## 1 INTRODUCTION

Nonalcoholic fatty liver disease (NAFLD) is the most common chronic liver disease worldwide. NAFLD is characterized by excessive lipid accumulation within hepatocytes in the absence of excessive alcohol intake or other known causes of steatosis such as hepatic viral infections and drugs (Loomba & Sanyal, 2013; Younossi et al., 2016). The histologic spectrum of NAFLD includes micro‐ or macro‐vesicular steatosis and steatohepatitis which can progress toward hepatic fibrosis and cirrhosis. NAFLD afflicts about a quarter of the general population, and its prevalence has increased dramatically in parallel with the obesity epidemic. Cardiovascular diseases (CVDs) are the leading cause of premature death in patients with NAFLD. Emerging evidence suggests that NAFLD is an independent risk factor for CVDs and most likely has a crucial role in the cardiovascular sequelae of metabolic syndrome.

Low‐grade inflammation is known to play a role in NAFLD, cardiovascular disease, hypertension, and some cancers (Rodríguez‐Hernández et al., 2013). The liver is the center for the production of high‐sensitivity C‐reactive protein (hs‐CRP) but seems also to be produced in the adipose tissue (Anty et al., 2006). Several studies reported that the hs‐CRP level, which is a known CVD risk factor, is correlated with liver histology in NAFLD patients (Kumar et al., 2020; Lee et al., 2017). It has suggested that hs‐CRP is an obesity‐independent marker of NAFLD (Genc et al., 2013; Nigam et al., 2013). Moreover, a recent meta‐analysis showed that about 40% of individuals with NAFLD had high arterial blood pressure (Younossi et al., 2016). Hypertension (HTN) is a serious medical condition that can lead to chronic renal disease, stroke, retinopathy, and heart failure. The precise mechanism of the initiation and development of HTN in patients with NAFLD is still uncertain, but it is thought to be mainly related to endothelial dysfunction and structural alterations in the tunica media layer of the arterial wall (Francque et al., 2016). Thus, individuals with NAFLD should constantly monitor their blood pressure and require advice about blood pressure control. HTN is a multifactorial disease that involves interaction between genes and environmental factors, particular diet.

Ample evidence exists regarding the important role of dietary modification on inflammation and blood pressure control. For example, adherence to a healthy dietary pattern such as the “Mediterranean” and “DASH” diet that emphasizes whole grains, legumes, vegetables, colorful fruit, fish, legumes, and olive oil can reduce blood pressure in hypertensive patients (Francisco et al., 2020; Magriplis et al., 2020). Also, organosulfur compounds in garlic (*Allium* *sativum L*) such as allicin and s‐allylcysteine are thought to be the main bioactive compounds responsible for the management of blood pressure, inflammation, and hepatic steatosis (Soleimani et al., 2020). These compounds improve blood pressure and inflammation by inhibiting the angiotensin‐converting enzyme, and transcription factor NF‐κB (Moosavian et al., 2020; Xiong et al., 2015b). At present, based on our knowledge, no clinical evidence exists regarding the effect of garlic consumption on blood pressure, and hs‐CRP level among patients with NAFLD. Therefore, we decided to implement this research to provide information in this respect.

## 2 METHODS AND MATERIALS

### 2.1 Study design and participants

This double‐blind, randomized, controlled trial was designed to ascertain the efficacy of garlic in patients with ultrasound‐diagnosed NAFLD. Participants were recruited from consecutive outpatients referring to the Metabolic Liver Disease Research Center at Isfahan University of Medical Sciences, Isfahan, Iran. Each study arm was needed 55 patients to detect a difference of 0.6 standard deviation (*SD*) in the Mean Arterial Pressure (MAP) response to garlic intake with an anticipated dropout rate of 20% and power 80% at a significance level of 5% (two‐tailed) (Allen Jr, 2011; Ried et al., 2018)

110 patients were selected after screening based on the inclusion and exclusion criteria. The inclusion criteria were as follows: adults aged between 20 and 70 years, new case‐patients with ultrasound technique‐proven fatty liver, serum glutamic‐oxaloacetic transaminase (SGOT) and serum glutamic‐pyruvic transaminase (SGPT) levels equal or over 40 IU/L, and stable blood pressure control by diet regimen or/and a stable dose of antihypertensive medication in the last 3 months. The exclusion criteria also were as follows: smoker patients, prior history of adverse reaction to garlic, pregnancy or lactation, other known causes of hepatic steatosis including alcohol intake, viral infections, and hepatotoxic drugs, medical history of endocrine diseases, renal disease, stroke, heart failure, hemochromatosis, Wilson's disease, and cirrhosis, or regular consumption of garlic. Patients were withdrawn from study participation when they become pregnant or need to any change their drugs.

The trial was conducted following the principles of the Helsinki Declaration. Each individual was informed of the overall objectives and procedures of this trial before obtaining the written informed consent. The study protocol was approved by a research ethics committee at Isfahan University Medical Sciences and then was registered at the Iranian Registry of Clinical Trials with the identifier number IRCT2014110819853N1.

### 2.2 Randomization and trial groups

Eligible participants were stratified according to sex and degree of fatty liver diagnosed by ultrasonography and then randomly allocated (in a 1:1 ratio) to receive 400 mg garlic tablet (enteric‐coated tablet containing 1.5 mg Allicin, Amin Pharmaceuticals, Isfahan, Iran) or placebo (enteric‐coated tablet containing 400 mg microcrystalline cellulose; School of Pharmacy, Isfahan University of Medical Sciences) two times a day for 15 weeks. Each garlic tablet contained dried garlic powder, aerosil, magnesium stearate, and croscarmellose sodium. Placebo tablets were quite similar to garlic tablets by the means of shape, size, color, taste, and smell. This dosage was chosen based on the previous studies conducted on the blood pressure and hs‐CRP (Mahdavi‐Roshan et al., 2017; Ziaei et al., 2001). The trial‐group assignments were concealed using sequentially numbered containers from all participants and investigators except the pharmacist who prepared randomization sequences generated with the use of a random‐numbers table.

### 2.3 Blood pressure measurements

A trained physician measured systolic blood pressures (SBP) and diastolic blood pressure (DBP) to the nearest 2 mm Hg using a calibrated mercury sphygmomanometer at the beginning and the end of the intervention. In each visit, measurements were implemented three times, at the two‐minute interval, on the left arm, with the patient in a sitting comfortable position, after 10 min rest, in the morning time (8–9 a.m.). The mean of three measurements was reported as the blood pressure values. The Mean Arterial Pressure (MAP) was calculated using the following equation: MAP = (SBP + 2DBP)/3.

### 2.4 Biochemical assessment

After 10–12 hr fasting condition, a venous blood sample was taken at baseline and after 15 weeks. Blood samples were immediately centrifuged at 3000 *g* for 10 min to separate serum and stored at −80°C until bioanalysis. Serum hs‐CRP was measured by the relevant ELISA kit.

#### 2.4.1 Confounding factors measurements

Weight was measured using a digital Seca scale (Saca 831), with light clothing, to the nearest 100 grams. Height was measured using a portable stadiometer (Seca) in the standing position to the nearest 0.5 centimeters. Then, Body Mass Index (BMI) was calculated by dividing weight in kilograms by height in meters squared. Waist circumference was measured without clothing at a level midway between the lower rib margin and the iliac crest at the end of exhalation and standing position using a tape, to the nearest 0.5 centimeters.

The change in dietary intakes was measured using a three‐day food record at the beginning and the end of the intervention. A trained nutritionist instructed patients on how to complete their food records. The amounts of each food item were converted to gram/day using Iranian Household Measures and then converted to the value of nutrients and calorie using Nutritionist IV software (First Data bank Inc., Hearst Corp).

Physical activity was measured using the international physical activity questionnaire (IPAQ) at the beginning and the end of the intervention. IPAQ is an instrument designed for population surveillance of physical activity among adults [15]. The Farsi‐translated version of IPAQ was validated among the Iranian population. The physical activity of the participants was calculated as metabolic equivalents (MET)‐minutes/week. According to the guidelines for data processing and analysis of the IPAQ, participants were stratified into three categories (low, moderate, and high levels of physical activity).

### 2.5 Statistical analysis

Data were analyzed using the Statistical Package for the Social Sciences (SPSS) software version 16 (SPSS Inc). The Kolmogorov–Smirnov test was used to examine the normal distribution of variables. A within‐group comparison was done with the use of the Paired Sample *t* *test* and Wilcoxon test. Independent Student's *t* test, Mann–Whitney *U* test, and chi‐square test were also used to ascertain differences in characteristics between two groups. We adjusted results by using the Analysis of Covariance (ANCOVA) test with baseline value, age, the grade of fatty liver, and change in energy intake, BMI, WC, and physical activity as covariates. Pearson correlation coefficients were analyzed to determine the inter‐variable relationships.

## 3 RESULTS

**FIGURE 1:**
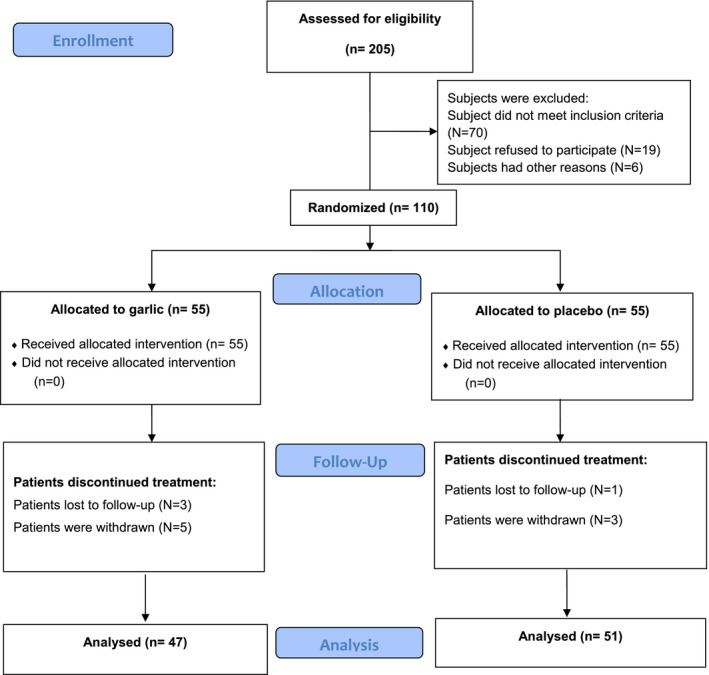
Flow diagram of the study based on CONSORT statement

A total of 205 patients were enrolled and underwent screening to participate in the trial; of whom 110 eligible patients were selected and allocated to receive either garlic tablet or a placebo tablet. Twelve patients were excluded from data analyses as follows: in the garlic group, 3 patients were lost to follow‐up, and 5 patients were withdrawn from the study; in the placebo group, 1 patient was lost to follow‐up, and 3 patients were withdrawn from the study (Figure 1). The two groups did not differ significantly in the dropout rates (14.5% vs. 7.2%; *p* = .22).

The participant's mean ( ± *SD*) age was 45.2 ± 11.6 years (range, 20–68), weight was 80.8 ± 14.6 kg (range, 53.2–117.7), and BMI was 29.5 ± 6.1 kg/m2 (range, 16.4–47.6). The majority of patients were female (59.2%); 72.5% had previous cardiovascular diseases, 52% had metabolic syndrome, and only 43.9% of patients used antihypertensive medication in which diuretics (65.1%) were more frequent drug use. Thirty‐seven patients (37.8%) had mild fatty liver; 54 patients (55.1%) had moderate fatty liver, and 7 patients (7.1%) had severed fatty on ultrasound imaging. Table 1 illustrates the baseline characteristics of patients. There were no significant differences in the primary characteristics of the patients in the two groups. Dietary intake was evaluated and indicated no significant differences between groups at baseline and the end of the study **(**Table 2 **)**. However, the mean change of body weight was statistically significant in the garlic group compared to the placebo group (−1.98 ± 2.09 vs. −0.83 ± 2.08; *p* = .010).

**TABLE 1:** General characteristics of study participants

Index	Garlic group (*N* = 47)	Placebo group(*N* = 51)	*p*‐value
Age (year)	46.4 ± 11.3	44.1 ± 11.8	0.32
Weight (kg)	82.4 ± 14	80.2 ± 15.1	0.42
BMI (Kg/m)	30.7 ± 5.3	28.5 ± 6.6	0.07
WC (cm)	95.2 ± 10.7	93.7 ± 9.2	0.1
Sex			
Male (*n* %)	40.4	41.1	0.94
Female (*n* %)	59.6	58.9	
NAFLD grade			
Mild (*n* %)	29.7	45	0.29
Moderate (*n* %)	61.7	49	
Severe (*n* %)	8.6	6	
SBP (mmHg)	13.3 ± 1.2	13.5 ± 1.2	0.5
DBP (mmHg)	8.9 ± 1.1	9.0 ± 0.8	0.1
MAP (mmHg)	10.3 ± 1.0	10.4 ± 0.9	0.4
Hypertension			
Pre (*n* %)	27	28	0.41
Stage 1 (*n* %)	12	18	
Stage 2 (*n* %)	8	5	
Antihypertensive drugs			
Diuretics (*n* %)	11	17	0.58
CCB (*n* %)	5	4	
ACE inhibitors (*n* %)	4	2	
Physical activity			
Low	65.9	60.7	0.65
Moderate	25.5	33.3	
High	8.6	6	

Note

Prehypertension: 80 < DBP < 90 OR 120 < SBP < 140; Stage 1 Hypertension: 90 ≤ DBP < 100 OR 140 ≤ SBP < 160; Stage 2 Hypertension: DBP ≥ 100‬ OR SBP ≥ 160. Values are expressed as mean ± *SD* for quantitative variables, and frequency (%) for qualitative variables. *p*‐values were calculated by Chi‐square test for qualitative variables and Independent‐sample *t* test for quantitative variables.

Abbreviations: ACE, Angiotensin‐converting enzymeNAFLD, Nonalcoholic fatty liver disease; BMI, Body mass index; CCB, Calcium channel blockers; DBP, Diastolic Blood Pressure; MAP, Mean Arterial Pressure; SBP, Systolic Blood Pressure; WC, Waist Circumference.

**TABLE 2:** Energy and macronutrient intake in the two groups at baseline and end of the study

	Garlic group (*N* = 47)	Placebo group(*N* = 51)	*p*‐value
Energy (kcal)			
Baseline	2,175.98 ± 410.51	2,301.32 ± 358.92	0.11
End	2,245.22 ± 366.26	2,136 ± 337.20	0.13
Carbohydrates (g/day)			
Baseline	304.63 ± 57.47	291.87 ± 47.61	0.68
End	322.18 ± 50.24	278.02 ± 43.83	0.81
Protein (g/day)			
Baseline	81.59 ± 15.39	101.03 ± 16.48	0.26
End	86.29 ± 13.45	96.23 ± 15.17	0.34
Fat (g/day)			
Baseline	70.11 ± 13.22	76.84 ± 12.20	0.14
End	74.15 ± 11.56	71.28 ± 11.24	0.25

Note

Values are expressed as mean ± *SD*. *p*‐values were calculated by Independent‐sample *t* test.

Multivariable‐adjusted mean change in blood pressure variables from baseline to 15 weeks intervention is shown in Table 3. The mean SBP significantly reduced (mean: −6.74; 95%CI: −9.18 to −4.31 mm Hg) in the garlic group, whereas no significant change was seen in the placebo group from baseline to 15 weeks (mean: 1.15, 95%CI: −1.21 to 3.52 mm Hg). Garlic treatment significantly reduced the SBP compared with the placebo, independent of potential confounding factors (mean: −7.89; 95%CI: −11.39 to −4.39 mm Hg). A significant reduction was seen in the mean of DBP (mean: −4; 95%CI: −5.68 to −2.34 mm Hg), while there was no significant change in the placebo group after 15 weeks of follow‐up (mean: 1.37; 95%CI: −0.25 to 2.99 mm Hg). Garlic treatment significantly reduced the DBP compared with placebo, independent of potential confounding factors (mean: −5.38; 95%CI: −7.77 to −3 mm Hg). The mean of MAP decreased significantly in the garlic group after 15 weeks of intervention (mean: −4.8; 95%CI: −6.5 to −3.1 mm Hg), while no significant change was seen in the placebo group (mean: 1.2; 95%CI: −0.48 to 2.8 mm Hg). Among dependent risk factors, garlic treatment reduced MAP by 6 mm Hg (95%CI: −8.4 to −3.6) compared with placebo after the 15 week intervention.

**TABLE 3:** Changes in blood pressure from baseline to 15 weeks of follow‐up

Variables	Mean Change ( ± SE)		Mean Effect size ( ± SE)	
	Garlic group (*N* = 47)	Placebo group (*N* = 51)	Garlic versus Placebo	*p*‐value
SBP, mm Hg	‒6.74 ± 1.25	1.15 ± 1.19	−7.89 ± 1.76	<.001
DBP, mm Hg	‒4 ± 0.84	1.37 ± 0.81	−5.38 ± 1.2	<.001
MAP, mm Hg	−4.81 ± 0.85	1.19 ± 0.82	−6.01 ± 1.22	<.001

Note

The mean change was adjusted for baseline value, age, grade of fatty liver, energy intake, BMI, WC, and physical activity.

Abbreviations: DBP, Diastolic blood pressure; MAP, Mean arterial pressure; SB, Systolic blood pressure.

**FIGURE 2:**
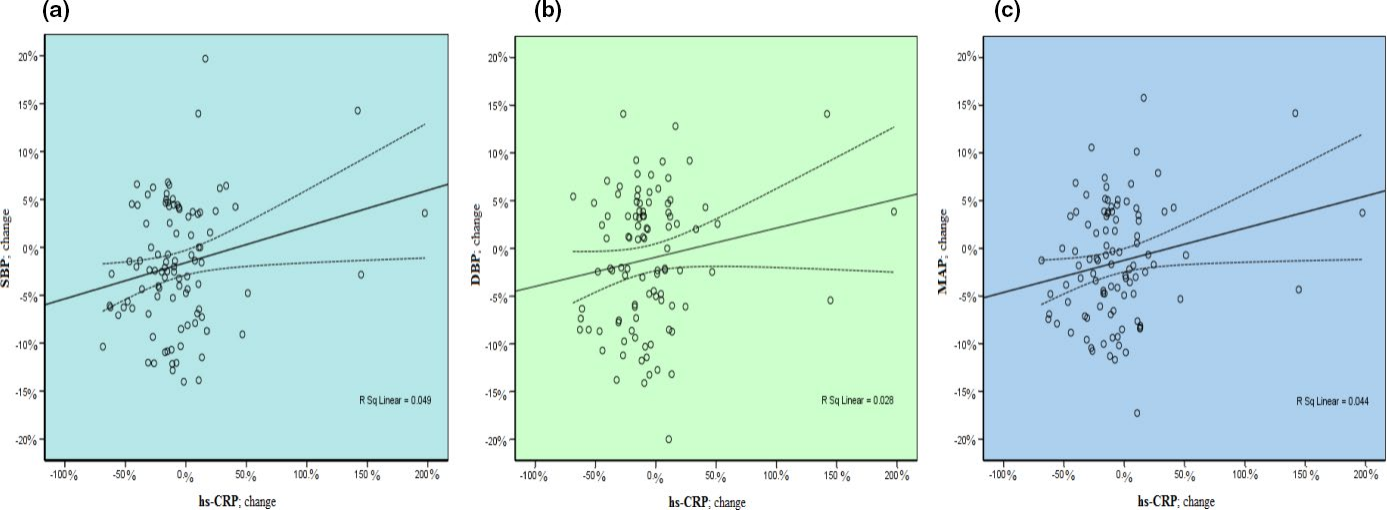
Association between percentage change in hs‐CRP and percentage changes in SBP (**a**), DBP (b), and MAP (**c**)

The mean ( ± *SD*) hs‐CRP concentration was 1.67 ± 0.52 mg/L in the garlic group and 1.62 ± 0.72 mg/L in the placebo group at the beginning of the trial. The two groups did not differ significantly in the serum concentration of hs‐CRP (*p* = .76). The mean ( ± SE) of percentage change in hs‐CRP concentration from baseline to 15 weeks of the intervention was −14.35 ± 0.58 and 1.75 ± 5.69 in the garlic and placebo groups, respectively. After adjusting the potential confounding factors including sex, age, BMI, physical activity, energy intake, weight change, and fatty liver grade, the percentage of reduced hs‐CRP was significantly higher in the garlic group compared with the placebo group (mean: −16.1; 95%CI: −32.7 to −0.53; *p* = .035). We also observed a significant positive relationship between percentage changes in hs‐CRP and percentage changes in SBP (*r* = 0.221; *p* = .029), DBP (*r* = 0.166; *p* = .012), and MAP (*r* = 0.210; *p* = .038) (Figure 2). No side effects were reported by patients during the intervention period.

## 4 DISCUSSION

The current study showed that the administration of garlic supplement (Allium sativum) to patients with NAFLD can reduce the blood pressure and the risk of CVDs independent of potential risk factors. We also found a positive relationship between change in hs‐CRP and blood pressure. To the best of our knowledge, no attempts were made to determine the effect of garlic on blood pressure and hs‐CRP in patients with NAFLD.

Our results showed that garlic intake can reduce SBP, DBP, and MAP in patients with NAFLD independent of potential risk factors. These findings are consistent with recent meta‐analysis reports. Xiong et al. reported that the administration of garlic powder can reduce SBP by 6.7 mm Hg and DBP by 4.8 mm Hg in hypertensive patients (Xiong et al., 2015a). Another meta‐analysis study in subjects with and without hypertension revealed that garlic intake can reduce the SBP by 16.3 mm Hg and DBP by 9.3 mm Hg in patients with hypertension, while it did not alter the SBP and DBP in patients without hypertension (Reinhart et al., 2008). Furthermore, regression analysis indicated a positive relationship between baseline values of blood pressure and reduction in blood pressure after the administration of garlic (Ried et al., 2008)). Two main mechanisms of action for the BP‐lowering properties of compounds in garlic have been offered, including the blockage of the Angiotensin‐II pathway and regulation of endothelial‐dependent vasodilation through intracellular hydrogen sulfide (H2S) and nitric oxide (NO) production (Ried and Fakler, 2014). Likewise, we found that reduction in blood pressure was parallel with the reduction in hs‐CRP, as a systemic inflammation marker produced in the liver. This finding is consistent with the previous study that showed a positive association between blood pressure and serum concentration of hs‐CRP in hypertensive patients (Dar et al., 2010). Thus, the BP‐lowering of garlic might be partially mediated by its well‐documented anti‐inflammatory properties.

It is well documented that chronic inflammation has central roles in the pathogenesis of CVDs. In clinical practice, serum concentration of hs‐CRP has been used to reliably predict the CVDs risk (Danesh et al., 2004; Ridker et al., 2000). Therefore, the current trial showed that the garlic treatment can contribute to reducing the risk of CVDs in patients with NAFLD independent of potential risk factors. Our findings are consistent with a recent meta‐analysis study in which the administration of garlic significantly decreased serum concentrations of hs‐CRP by 0.82 mg/Dl (Taghizadeh et al., 2019). These findings are worth noting because cardiovascular events are the leading cause of premature death in patients with NAFLD. Likewise, emerging evidence suggests that NAFLD is an independent risk factor for CVDs and most likely has a crucial role in the cardiovascular sequelae of metabolic syndrome. Several pathological conditions are involved in the development of CVD in patients with NAFLD, including systemic inflammation, oxidative stress, endothelial dysfunction (NO), and structural alterations in the arterial wall (Francque et al., 2016). Garlic mainly contains organosulfur compounds with antioxidant, anti‐inflammatory, and BP‐lowering properties (Iciek et al., 2009). Garlic and its constituents have been shown to inhibit the activation of NF‐kB as the main transcription factor for many inflammatory mediators, such as TNF‐α, IL‐1, IL‐6, CXCL1, CXCL2, ICAM‐1, and VCAM‐1, in response to various.

stimuli through the inactivation of JNK, p38 MAPK, and ERK pathways (Bauer et al., 2015; Keiss et al., 2003; Kim et al., 2013).

The main limitation of the current trial is a small number of patients having a severe grade of fatty liver, which can affect its generalizability to this group. However, this trial is strengthened by stratified randomization, well‐matched baseline values, low dropout rate, high compliance rate to treatment, and adjusting the results for potential confounding factors. Further research is needed to compare antihypertensive drugs with garlic powder among NAFLD subjects for choosing the best intervention to control hypertension. It would also be important to assess the effect of garlic supplement on other cardiovascular diseases in patients with NAFLD and to compare it with another drug therapy strategy.

## 5 CONCLUSION

In conclusion, our present trial indicates that garlic supplementation can be useful to reduce blood pressure and the risk of cardiovascular disorders in patients with NAFLD.

## CONFLICT OF INTEREST

The authors declare that they have no competing interests.

## AUTHOR CONTRIBUTION

**Davood Soleimani:** Data curation (lead); Writing‐original draft (lead). **Seyedeh Parisa Moosavian:** Writing‐original draft (equal). **Hamid** **Zolfaghari:** Formal analysis (lead). **zamzam paknahad:** Conceptualization (equal).

## ETHICAL STATEMENTS

This study was conducted according to the guidelines laid down in the Declaration of Helsinki, and all procedures involving research study participants were approved by the research ethics committee at Isfahan University Medical Sciences and then was registered at the Iranian Registry of Clinical Trials with the identifier number IRCT2014110819853N1. Written informed consent was obtained from all study participants.

## Data Availability

The datasets generated or analyzed during the current study are not publicly available but are available from the corresponding author on reasonable request.
